# Challenges that healthcare practitioners experience in the comprehensive assessment of patients with non-communicable diseases: a preliminary investigation

**DOI:** 10.4314/ahs.v21i3.39

**Published:** 2021-09

**Authors:** Lynn Smith, Heather Morris-Eyton, Habib Noorbhai, Yoga Coopoo

**Affiliations:** University of Johannesburg, Faculty of Health Science, Department of Sport and Movement Studies

**Keywords:** Healthcare practitioners, non-communicable diseases, qualitative research

## Abstract

**Background:**

Resource allocation and access to comprehensive treatment in the public healthcare sector are always under pressure. This pressure takes the form of staff shortages, treatment models and the holistic care of patients, compromising basic healthcare in South Africa.

**Objectives:**

The study's aim was to determine the challenges that healthcare practitioners experience while assessing patients with non-communicable diseases, in private and public healthcare sectors in the Gauteng Province of South Africa.

**Methods:**

The research design was exploratory and contextual. Qualitative data were collected through focus groups and semi-structured interviews among healthcare practitioners (n = 12). Data analysis took place using Atlas.ti 8.4 Windows (2020). Inter-rater reliability (r = 93.68%) was calculated to ensure the rigour and validity of the results.

**Results:**

From the discussion, four themes emerged: 1) limited consultation time; 2) overwhelming economic impact and healthcare cost for facilities and patients; 3) holistic patient care encompassing physical, mental and socioeconomic components; and 4) lack of patient education due to time constraints experienced by healthcare practitioners.

**Conclusion:**

Consultation times are reduced due to a shortage of medical staff, patient numbers, equipment, and poor working conditions. By improving these conditions, patients across all socioeconomic groups will be better assisted, treated, and educated, benefiting from equal access and quality healthcare.

## Introduction

The social determinants of health encompass the conditions that individuals are exposed to throughout life and how these conditions contribute to their health status.^1^ In the South African context, the social determinants result in health inequities, resulting in the quadruple burden of disease and a high incidence of premature mortality.^2^ It is estimated that non-communicable diseases (NCDs) account for approximately 51% of all deaths in South Africa.^3^ The distribution of NCDs illustrates the socio-economic disparities in the country, with the heaviest burden being among the poorer communities.^4^ One of the targets of the third United Nations Sustainable Development Goals is to reduce premature mortality by preventing and treating NCDs, and promoting mental health and well-being.^5^ This target is a key priority of the global NCD action plan. Historically, the prevention and treatment of NCDs was largely neglected in South Africa due to the overwhelming prevalence of communicable diseases such as human immunodeficiency virus (HIV), autoimmune deficiency virus (AIDS) and tuberculosis. However, the shifting of disease burden from communicable disease to NCDs has placed pressure on healthcare systems that are already under-resourced and under-funded.^6^,^7^ The quality of healthcare in South Africa has been compromised due to prolonged waiting times, shortage of human resources, inefficiency, high cost and unequal distribution of resources, to name a few factors.^8^

According to the White Paper on the National Health Insurance (NHI) by the Department of Health in South Africa,^9^ the NHI aims to be a people-centred healthcare service, which should ultimately improve patient satisfaction and result in better health outcomes and quality of life for patients across all socio-economic groups. It also aims to improve the quality of life of healthcare practitioners based on the assumption that the provision of basic social infrastructure and amenities will recruit satisfied and motivated staff, ensuring better productivity and retention.^9^ The current public healthcare system, catering for patients without medical aid, accounts for approximately 82 out of every 100 South Africans.^2^ These government-funded healthcare facilities are inundated with patients and have shortages of staff, funding, equipment, and essential medicines, a situation that is currently exacerbated by the Coronavirus pandemic. Large patient numbers have resulted in lengthy waiting times and shortened consultations with healthcare practitioners. Although there are currently no guidelines on what the ideal length of a consultation should be, research indicates that patients would prefer additional time with their healthcare practitioner. 10,11

A study conducted in the Free State and Gauteng provinces of South Africa linked shorter waiting times to patient satisfaction, and patient satisfaction to better treatment adherence and health outcomes.^12^ In addition, access and affordability of healthcare and the impact on the economy has been identified as another challenge within the current system. Not only does the NCD epidemic in South Africa entail unaffordable costs for the patient, but the families of the deceased patient are also left with the financial burden of losing a breadwinner, and employers spend more for paid time off work.^13^ Furthermore, the prevalence of NCDs has increased among the working-age population, impacting the productivity and ultimately the economy of the country.^13^ To optimise treatment, health outcomes, and government resources, a holistic approach to patient care should be observed. Holistic patient care is intended to provide a healthcare practitioner with a comprehensive understanding of their patient, as well as their various needs for care which may have an impact on healthcare systems.^14^ It has been proposed that future research studies explore the various impacts that culture, family structure and social support can have among older patients living in low- to middle-income countries, particularly in Sub-Saharan Africa, as these factors can cause morbidity and lower quality of life.^15^ Education should be at the forefront of treatment with the purpose of encouraging patients to take responsibility for the management of their condition(s) and health. A South African study advocating behavioural change counselling revealed the importance of providing comprehensive patient education and counselling to patients with chronic disease, starting at the time of their diagnosis.^16^ Regrettably, hypertensive and diabetic patients have reported poor knowledge about their condition and the role of lifestyle modifications in the management thereof. This has been linked to poor decisionmaking about their treatment, lack of autonomous support, and poor sense of control over health-related decisions, all resulting in low levels of self-care and self-management in the long-term.^17^

In line with international best practice, South Africa has made significant progress in the attempt to reduce the prevalence of NCDs by implementing policies to decrease tobacco and alcohol consumption, and interventions that address poor nutrition and physical inactivity. 4,18 However, much improvement is needed in the integration of primary healthcare services, human resources and financial sectors, which affects the impact of policy implementation on the prevention and treatment of NCDs. Moreover, there is a shortage of healthcare practitioners and finance for sustainability, particularly in rural areas, resulting in healthcare inequality. As a result, the aim of this paper was to gain insight into the perceptions and experiences of healthcare practitioners regarding the challenges of comprehensively and holistically assessing patients with NCDs.

## Methodology

### Study design

This research study adopted an exploratory, contextual study design approach in which qualitative data was collected. The Delphi method was employed during this study to obtain consensus from an expert panel, using a controlled method of feedback.^19^

### Study population

An expert panel (n = 12) of various healthcare practitioners was invited to participate, with the aim of gaining insight into their perceptions regarding the challenges they experience when assessing overall patients with NCDs. The panel included two healthcare practitioners from each of the following professions: general practice, physiotherapy, biokinetics, occupational therapy, clinical psychology and dietetics. This range of healthcare practitioners was chosen with the aim of obtaining a broad perspective of factors that can contribute to overcoming the barriers associated with NCDs. Within each profession, one healthcare practitioner practised in the private sector, whereas the other practitioner had experience only in the public healthcare sector.

### Data collection

Focus groups and semi-structured interviews were conducted with the expert panel (E1-E12) to identify challenges experienced during consultations. In this study, the twelve experts were randomly assigned to focus groups. In instances where practitioners were not able to join a focus group session, semi-structured interviews were conducted. The same moderator was present in each session to ensure consistency. Both the focus groups and semi-structured interviews were interactive in nature, and a standard set of guide questions was used to start discussions. However, the researcher altered the sequence of questions and probed for information from respondents by making use of subsidiary questions.^20^ All interactions were audio recorded with consent and the responses were manually transcribed by the researcher. These were sent to two random experts for member checking, which strengthened the validity and reliability of the data collected.

### Data analysis

This research made use of first and second cycle coding processes. First cycle coding involved in-vivo coding whilst the second coding process involved descriptive coding. Atlas.ti 8.4 Windows (2020) was utilised for the coding of the data. A co-coder was also recruited to reduce perspective error and ensure reliability of the coding process. A confidentiality agreement was entered into between the researcher and the co-coder to verify the codes.

An inter-rater reliability test was done to ensure scientific rigour, validity and reproducibility of the data analysed, as well as to reduce perspective error.^21^ Reliability between the researcher and co-coder was assessed (r = 93.68%).

The physical, mental and socio-economic components are regarded as the foundational pyramid of health-related quality of life (HRQoL).^22^ Age, gender, race, education level, employment status, and the size of a patient's household are all determinants of the very high rate of health expenditure and impoverishment, leading to unaffordable healthcare, particularly for those attending public health facilities.^23^ Subsequent to the focus groups and semi-structured interviews in this study, the physical, mental and socio-economic components were further delineated.

### Ethical considerations

To ensure ethical practice, the research was approved by the Research Ethics Committee of the relevant institution (REC-01-04-2018). Prior to participation in the focus groups and interviews, participants were given information letters describing the study. If they were willing to participate, an informed consent form was signed.

## Results and discussion

The main aim of the focus group discussions and semi-structured interviews was to gain insight into the challenges that healthcare practitioners face when assessing patients with NCDs. From the identified challenges, four themes emerged: 1) consultation time, 2) economic impact and healthcare cost, 3) holistic patient care and 4) patient education. These themes will be the focus of the following results and discussion sections.

### Consultation time

Time constraints is a limiting factor when assessing all relevant factors associated with NCDs. The standard perception of healthcare practitioners and patients worldwide is that consultation times are too brief.^24^ This is related to the large numbers of patients to be seen on a daily basis. For patients, the time spent in the waiting room is lengthy, and in some cases, more than one day. However, the actual time of attention is brief and insufficient, which compromises the quality of medical care.^24^ During a focus group discussion, one of the general practitioners (E5) said: ‘*There isn't very much time to actually concentrate on all of that, which I feel is a tragedy, because it's a patient as a whole, not just their physical or clinical problems.’* Research in Malaysia found that longer consultations are more likely to result in doctors identifying psychosocial issues, prescribing less mediation, and offering more preventative advice.^11^ Behaviour change education will play an important role in many NCD conditions. Managed care in clinics has been blamed for shorter patient visits, as administrative tasks and economic impact have taken precedence.^25^ Another doctor (E1) practising in general practice stated during the interview: *‘I really try and look at patient's circumstances, but it really is a challenge in terms of time. Because if I want to spend at least 15 minutes with a patient I'm never going to get done*.’ A systematic review of 67 countries examining the relationship between consultation length and organisational-level economics, on the one hand, and health outcomes, on the other, concluded that there is a large variance in consultation length on a global level and that some physicians are spending only 48 seconds with their patients.^26^ Furthermore, 50% of the global population reported spending less than five minutes with a primary care physician.^26^ Brief consultations such as these are likely to have an adverse effect on patient healthcare, as well as on physician workload and stress.^26^ In a study conducted in Sweden, patients have reported that general practitioners who focus only on the actual health problem were stressed and rushed during a consultation.^27^ There is also a reported association between short consultation time and physician burnout, as doctors feel a lack of personal accomplishment as a result of being less competent at managing multimorbid patients.^26^ Furthermore, it has been reported that physicians' level of job satisfaction is linked to patient relationships, and the main reason for dissatisfaction was time pressures.^25^ It would seem prudent for South African universities to train more medical doctors to serve a large and ever-expanding population.

### Economic impact and healthcare cost

Currently, only 16% of the South African population belong to medical aid schemes, implying that the majority of the population rely on the public healthcare system for treatment.^8^ In this study, the expert panel expressed the view that the financial burden on patients is a major challenge affecting their ability to adequately treat patients. A clinical psychologist (E8) working mainly in the public healthcare sector argued that patients cannot adhere to their treatment if they do not have sufficient finances: ‘*If you think of the financial domain in the South African context, it has a significant impact because even if you want to be treatment adherent, but you don't have the finances and the public system doesn't give you enough medication, then it has an impact on how you use them, or the disease, or your self-efficacy over time*.’ As a result, there are many cases of non-adherence to medication, of which the most cited reasons are distance from clinics or hospitals and medication costs.^28^ Despite reforms intended to promote equal access to healthcare in the post-apartheid era, South Africa still falls short on availability and affordability, particularly in the poorer and rural communities, mainly due to the country's deficient transport systems.^29^ A study conducted across six middle-income countries found that a considerable percentage of out-of-pocket expenditure among South African patients with multimorbid conditions was spent on transport costs.^30^ Similarly, a study conducted among diabetic patients in two public hospitals in Tshwane, South Africa, calculated the patients' contribution to transport costs to be over 50% of their total healthcare costs.^23^ This exceeds the 10% threshold of the households' capacity to pay, which is classified as a catastrophic health expenditure. 23 One of the physiotherapists (E9) interviewed stated that: ‘*In these economic times to be honest, I think your financial situation plays a big role in a lot of people's lives*.’ Although more than half of all doctor consultations in South Africa are at no charge to the patient, and primary care is provided free of charge, studies reveal that in 2017 the economic impact of poor healthcare resulted in an increase in total healthcare costs by more than 80%.^30^,^28^ In South Africa, financial cost is a well-known factor for poor access to adequate healthcare, and income inequality, poverty and unemployment are among the highest in the world, which is a large contributor of the inequalities in the distribution of health.^31^,^32^ This was echoed by a dietician (E2) who believes that: ‘*adding the social component to a consultation is very important, especially if you want to roll it out in our country, because, our country is so diverse and you have these two polls of affluence. Majority are not able to afford private healthcare*.’ The NHI aims to resolve these disparities by providing funding and access to quality and affordable healthcare based on patients' needs, regardless of socio-economic status.^9^ The challenges highlighted in this paper paints an unprecedented and concerning picture of the healthcare system in South Africa. The recent Coronavirus pandemic emphasises the need for vast improvements in order to equip the healthcare system to deal with such outbreaks in the future. In a country such as South Africa, where more than half of the country is rural in nature, it is imperative that the healthcare system places a clinic in every small rural village and many more in larger city centres. This would imply training more healthcare practitioners to service these clinics.

### Holistic patient care

Events occurring in early life have a profound impact on the development of NCDs and the effects of aging increase the complexity of the disease. By addressing the physical, social and emotional needs of a patient, patients will have a better understanding of the effects of an illness and become better equipped to deal with their diagnosis, thereby improving their lives and teaching them self-responsibility.^14^ In a study conducted among eight nurses with the aim of presenting a concept analysis of holistic care, nurses indicated that the patient's physical, social and mental needs are addressed, because the patient is respected, and treated as a unique person, therefore ensuring the patient's comfort is the primary objective.^14^ Another study that echoed this statement was conducted in Sweden among 10 participants with a variety of chronic conditions. This study highlighted that patients felt that they were ‘*treated as individuals with a focus on their whole person and not just their health problem*’.^27^ Given the emphasis placed on assessing the physical, mental and social needs of patients, the concept of holistic care for the purpose of this study was divided into these three categories. These three concepts are all interrelated and can have a profound impact on each other. Research shows that physical limitations can influence psychological stress, but with relatively high levels of perceived social support, individuals experience less psychological stress associated with their physical limitations.^33^

When asking healthcare practitioners about the first concept, which addressed physical limitations, their patients' experiences and how it impacts their overall HRQoL, a physiotherapist (E9) commented: ‘*You get those in the early stage of disease that have no limitation and then on the opposite end of your scale is severe limitations and because of that physical limitations, it affects every other part of their life. Also, the emotion comes into it ... the frustration*.’ A study conducted among veterans aged 25 years and older reported that there is a significant relationship between chronic conditions, physical limitations and mental health.^34^ However, research suggests that physical limitations may cause less psychological distress among older individuals because there is a general notion that limitations in activities of daily living tend to be greater with increasing age.^33^

The effects of stress and anxiety are likely to have an influence on the second aspect of holistic care – a patient's mental health. This underscores the need to assess mental health as an aspect of HRQoL. During interviews, a clinical psychologist (E12) working with patients in the private and public healthcare sectors emphasised that many patients battle to adjust to the holistic changes they need to make, due to the way they view their illness, also affecting their mental health and ability to get treatment overall: ‘*I think often patients forget to think multidisciplinary or holistically about their condition because a patient just came to me and I had to help her because it's a mental thing. She eats too much, and doesn't follow the diet she should be following, but I think it's so much more. So, I think this is a very important thing’*. In addition, a dietician (E2) noted the following in patients: ‘*We're seeing people that have been diagnosed with hypertension, and it's usually amongst younger people. They are completely stressed out about this diagnosis, that they cannot function normally. A lot of patient's mental capacity is blocking them from doing their everyday tasks*.’ The NCD-related complications place significant demands on individuals and families, causing stress and a negative impact on a patients' financial status, social welfare, productivity and health behaviours.^35^ Not only is poor mental health associated with greater disability, but patients suffering from mental health disorders are often less likely to seek treatment due to the associated stigma.^36^

The third aspect of holistic care refers to the social needs of patients. Research has proven that there is a direct link between loneliness, isolation, living alone and poorer mental health.^37^ In fact, the overall odds for mortality associated with social isolation are comparable to that of smoking, and exceed the risks associated with hypertension and obesity.^38^ In this study, the researcher felt it was important to assess the social networks of patients and what the experiences of the expert panel was in terms of overall HRQoL. A clinical psychologist (E8) commented: ‘*They isolate themselves because of their health, and that then goes hand in hand with alcohol use and substance abuse*.’ Patients with NCDs are 1.4 to 2.2 times more likely to develop anxiety, depression and a dependence on alcohol compared to their apparently healthy counterparts.^35^ In a medical evaluation, it is imperative to assess patients' social risk factors and highlight the importance that nurturing relationships can have on lifestyle and well-being, as social isolation contributes to depression, which reduces overall quality of life.^38^,^39^ During another interview, a physiotherapist (E11) linked loneliness with treatment adherence: ‘*So many people are living alone these days, and most of them don't even have anyone to monitor them and to see they're actually taking their medication*.’ A meta-analysis which identified longitudinal studies investigated loneliness and social isolation as possible risk factors in the development of cardiovascular disease, since it impacts on nutrition, physical activity, sleep, treatment adherence and cooperation, and biological markers.^38^ By considering a patient's social circumstances, effective referrals to mental health and social support services can also be put in place.^38^

During the interviews, the researcher mentioned concerns about including questions in the questionnaire that would possibly be breaching some aspects on other healthcare scopes of practice. This is how one of the clinical psychologists (E8) responded: ‘*I'm sure an occupational therapist asks some of those questions and as a biokineticist, you aren't diagnosing so it's completely fine to ask questions. It makes you a better healthcare practitioner if you ask questions*.’ A physiotherapist (E9) supported this approach to treatment by saying: ‘*If a patient can expand on the way it affects them, then you know it's a physical thing in terms of I need this aid or whatever. If they can't afford it or it's a mental, emotional or psychological aspect then they stress about it and the medical condition gets worse. It just gives you that direction to consider in the entire well-being*.’ Practitioners whose emphasis is on individually and holistically promoting health and wellness have been praised by patients worldwide.^27^ Besides the positive impact that addressing conditions holistically can have on the patient, this approach can also have a direct benefit on the healthcare practitioner. It has been proven that holistic care enables nurses to feel satisfied and useful, which promotes job satisfaction and an inclination to stay in the profession for longer.^14^ There is clear evidence illustrating the need for holistic interventions with a multidisciplinary approach to promote the lifespan of patients worldwide.^40^

### Patient education

Although the expert panel alluded to the importance of patient education as part of their treatment, one cited that time is a constraint, and another indicated that it is imperative to be perceptive of the patient's knowledge, in order to clarify in which direction to steer the discussion. A general practitioner (E1) working in a public healthcare setting highlighted this as a concern by saying: ‘*We don't have a dedicated dietician. It's like a health worker, and she's also not been well, so I really don't have the time to explain to each and every patient about the diet*.’ As a solution, a study among healthcare providers' perspectives of diet-related NCDs in South Africa suggested that educational talks delivered by dieticians to the hundreds of patients waiting at the clinic be offered as an affordable intervention.^41^ In addition to this, another study conducted among patients in Thailand with rheumatoid arthritis reported that providing an information pamphlet to patients, with or without direct counselling, can improve medication adherence.^42^ Another general practitioner (E5) indicated that: ‘*Often patients have misconceptions about symptoms pertaining to their illness. For example, hypertensive patients will say they have headaches attributed to hypertension. You then have to explain that hypertension is silent. So, it gives you an idea as to what is the concepts of the patients pertaining to the symptoms of the illness and that way you also teach*.’ In a study conducted among poorly controlled type two diabetes patients, patients were given education on correct insulin storage, injection techniques, injection site rotation, syringe used, dosing and timing, compliance with insulin and other anti-diabetic medications.^42^ This study yielded improvements in glycaemic control and quality of life when extensive education was delivered about the disease, adherence and self-care.^43^ However, a systematic review on coronary heart disease demonstrated that patient education alone does not reduce the mortality of cardiac patients, reoccurrence of cardiac events, the prevalence of bypass or angioplasty surgery, or admission into hospital as a result of cardiac disease.^44^ Their evidence confirmed that education based interventions yielded improvements in all domains of HRQoL, particularly the mental scores, and also demonstrated behaviour changes among patients.^44^ This supports the notion of offering comprehensive rehabilitation to cardiac patients, which includes education.^45^,^44^ In addition, a study consisting of interdisciplinary sessions on diet, behaviour change, education and support groups, in an attempt to reduce patients' weight, concluded that working in a multidisciplinary team would provide the patient with an adequate programme that will yield benefits for those willing to make behaviour changes.^46^

## Recommendations

During the data collection, it became apparent that there is a need to develop an integrated questionnaire for healthcare practitioners which will assess HRQoL encompassing physical, mental and socio-economic domains, and could be administered easily to patients. A HRQoL questionnaire may prove to be cost effective as it requires minimal resources and can be completed while in the waiting area of a healthcare facility. It will provide a better outcome for rehabilitation, as the health practitioner can easily identify the challenges facing the patient and ensure a corrective rehabilitation programme.

## Strengths and Limitations

The healthcare practitioners that formed part of the sample in this study represented an equal distribution of private and public healthcare experience. This information strengthens the research and enabled the researchers to obtain accurate results indicating disparities between private and public healthcare systems. Due to the relatively small sample size and the fact that this is a preliminary investigation, further studies should be conducted among larger groups of a more diverse range of healthcare practitioners. A better understanding of holistic patient care which infers adequate health-related quality of life in the South African context should be documented.

## Conclusion

The literature has highlighted the importance of including physical, mental and socio-economic domains in overall health assessments. The burden on the public healthcare system in South Africa stems from a shortage of medical staff, insufficient consultation time, and poor access for patients to have regular health screenings due to financial constraints and logistical challenges. All biokineticists and physiotherapists are trained to perform basic health screening on patients. Adding a biokineticist to the multidisciplinary primary healthcare team would assist in alleviating the current burdens experienced in hospitals and clinics across South Africa. Healthcare practitioners need to be better equipped to adequately assist, treat, and educate patients; therefore, training programmes and resources are an absolute necessity. Patients across all socio-economic groups must be given equal quality and access to healthcare by increasing facilities, competent staff, and essential medication and equipment. Understanding these challenges is vital for informing policy and equal distribution of resources, especially with the enactment of the National Health Insurance in South Africa.
